# Hydrocele and Hydro-Sarcocele

**Published:** 1874-03-15

**Authors:** A. Given

**Affiliations:** Louisville, Ky.


### HYDROCELE AND HYDRO-SARCOCELE.

BY A. GIVEN, M.D., LOUISVILLE, KY.

IN August, 1873, Mr. E., of New Orleans, aged fifty-five years, gave the following history of his case: Sometime during the winter of 1872 he noticed that the left side of the scrotum was enlarging. In April, 1873, he was operated on, and a large quantity of liquid was drawn off. In a few weeks after the operation the scrotum was as large as ever. He was again operated on, and over a pint of serum was drawn off, and a medicated liquid was injected, which caused much pain and inflammation; from which he suffered nine days.

In about two weeks after the last operation, the scrotum was re-filled. He refused to submit to another operation, and applied to me for treatment. I put him upon the use of the following:

℞—Camph. soap, liniment, ℥ v.

Tinct. iodine, ℥ j.

Glycerine, ℥ ss.

Mix, and apply to scrotum three times a day.

For internal treatment, he was directed to take twenty-five drops of the muriated tincture of iron, in half a glass of sweetened water, three times a day.

On February 5th, 1874, I received a letter from my patient, stating that, after using the medicines one week, the swelling began to subside. He continued the treatment two months, and might have discontinued it sooner, but was afraid the disease would return.

He closed by saying that he was cured, and that he had not lost a day's work during the treatment.

HYDRO-SARCOCELE.—Mr. W., aged forty years, says that one year ago he received a blow on the testicles, which caused inflammation of the left testicle. The acute symptoms passed off, and left the testicle enlarged and tender. About three months ago, he noticed that the scrotum was enlarging.

Upon examination, I noticed that the left testicle was about three times larger than the right one; and that it was quite painful to the touch; and the scrotum contained a large quantity of serum. The scrotum measured ten inches in circumference. I kept the parts at rest, and put him upon the same plan of treatment as above, and gave opium to relieve pain; and in four weeks he was able to return to his work. The testicle was free from tenderness, but was somewhat larger than the other; the scrotum contained no serum, that I could detect.

I have cured two cases of hydro-sarcocele, and eleven cases of hydrocele, eight in children and three in adults, by the above plan of treatment.

I need not dwell on the therapeutic action of iodine in these affections, for it is, undoubtedly, well understood by the profession.

In regard to the internal use of muriated tincture of iron in hydrocele, I imagine that it assists in the cure by increasing the plasticity of the blood, and thus retards the tendency to dropsical effusions; and, by improving the quality of the red globules of the blood, the parts are stimulated to a more active resorption of the effused serum.

---

BROMIDE OF CALCIUM.—This remedy, suggested by Dr. Hammond, has been investigated by Dr. Guttmann, of Berlin, whose paper appears in the *Allgemeine Medicinische Central-zeitung*, December 6th. The latter finds it about one-third or one-fourth as strong as the bromide of potassium, and disagrees entirely both with Dr. Hammond's clinical and chemical theories of its value.—*Phil. Med. and Surg. Reporter*.

